# RAAS Inhibitor Prescription and Hyperkalemia Event in Patients With Chronic Kidney Disease: A Single-Center Retrospective Study

**DOI:** 10.3389/fcvm.2022.824095

**Published:** 2022-02-11

**Authors:** Eleonora Riccio, Ivana Capuano, Pasquale Buonanno, Michele Andreucci, Michele Provenzano, Maria Amicone, Manuela Rizzo, Antonio Pisani

**Affiliations:** ^1^Institute for Biomedical Research and Innovation, National Research Council of Italy, Palermo, Italy; ^2^Department of Public Health, Chair of Nephrology, University Federico II of Naples, Campania, Italy; ^3^Department of Neurosciences, Reproductive and Odontostomatological Sciences, University of Naples Federico II, Naples, Italy; ^4^Department of Medical and Surgical Sciences-Renal Unit, “Magna Graecia” University, Catanzaro, Italy

**Keywords:** hypertension, chronic kidney disease, hyperkalemia, RAAS inhibitor, prevalence

## Abstract

Hyperkalemia is common in patients treated with renin–angiotensin–aldosterone system inhibitors (RAASis), and it represents the main cause of the large gap reported between guideline recommendations and real-world practice in chronic kidney disease (CKD). We conducted a CKD-population-based restrospective study to determine the prevalence of patients with CKD treated with RAASis, incidence of hyperkalemia in patients with CKD treated with RAASis, and proportion of patients with RAASi medication change after experiencing incident hyperkalemia. Among 809 patients with CKD analyzed, 556 (68.7%) were treated with RAASis, and RAASi prescription was greater in stages 2–4 of CKD. Hyperkalemia occurred in 9.2% of RAASi-treated patients, and the adjusted rate of hyperkalemia among patients with stage 4–5 CKD was 3-fold higher compared with patients with eGFR > 60 ml/min/1.73 m^2^. RAASi treatment was discontinued in 55.3% of the patients after hyperkalemia event (74.2% discontinued therapy, 3.2% received a reduced dose, and 22.6% reduced the number of RAASi drugs). This study shows that the incidence of hyperkalemia is frequently observed in patients with CKD patients with RAASis, and that rates increase with deteriorating levels of kidney function from stages 1 to 3. RAASi medication change following an episode of hyperkalemia occurred in almost half of the patients after experiencing hyperkalemia.

## Introduction

Chronic kidney disease (CKD), defined as decrease in kidney function manifested as presence of structural or functional kidney abnormalities or estimated glomerular filtration rate (eGFR) < 60 ml/min/1.73 m^2^, has a major effect on global health, both as a direct cause of global morbidity and mortality and as an important risk factor for cardiovascular disease (which is the leading cause of death in CKD). Blood pressure lowering therapy has shown to prevent the onset of poor cardiovascular outcomes and delay the progression of kidney disease (1, 2).

Several randomized clinical trials have clearly shown that inhibition of the renin-angiotensin aldosterone system (RAAS) can reduce the risk of death and slow disease progression in patients with heart failure (HF), CKD, and diabetes (3–7). Therefore, evidence-based treatment guidelines recommend the use of RAAS inhibitors (RAASis) as first-line blood pressure lowering therapy for patients with CKD and proteinuria, and diabetes and hypertension (8–10). Moreover, the guidelines specifically recommend the use of maximum tolerated dose of RAASis, since results of clinical trials demonstrated that best treatment benefits were obtained with moderate to high doses (8–10).

However, the use of these drugs may be limited, since they can cause hyperkalemia (11) (typically defined as serum potassium levels > 5.5 mmol/L), which can be further exacerbated when these drugs are used in combination (12).

Several recommendations for minimizing the risk of hyperkalemia have been provided by current treatment guidelines, including: avoiding RAASi therapy in patients at risk of hyperkalemia, discontinuing drugs that can interfere with renal potassium excretion before initiating RAAS inhibitors, titrating the dose of the RAASis, and performing regular monitoring of potassium serum levels (8–10). Moreover, if hyperkalemia develops after initiating therapy, it is recommended to discontinue or lower the dose of RAAS inhibitors (8–10).

Therefore, there is a substantial gap between recommendations in treatment guidelines and the real-world practice in the use of RAASis. This point is crucial, since discontinuation of RAASis after the onset of hyperkalemia may increases the risk of poor outcomes, with a combined deleterious effect of hyperkalemia and withdrawal of RAASi on prognosis (13).

Studies that have assessed whether hyperkalemia can affect subsequent RAASi treatment regimen are limited. Recent population-based studies are limited by the inclusion of relatively small proportions of patients with CKD (14) or according to renal function, (15) predominance of males in assessed cohorts (96%) (16), and small size [*n* = 238 (17) and 258 (18) patients].

Therefore, we conducted a CKD-population-based retrospective cohort study on adult patients with CKD to determine the prevalence of patients treated with RAASis and distribution of the use of RAASis in different CKD stages. Moreover, we evaluated the incidence of hyperkalemia in patients with CKD treated with RAASis, and proportion of patients with RAASi medication change after experiencing incident hyperkalemia.

## Methods

We conducted a retrospective, CKD-population-based cohort study using healthcare data from the medical records of patients with CKD referred to the Department of Nephrology of University “Federico II” of Naples from January 2010 to December 2019.

The study included all adult (≥ 18 years) patients presenting an estimated glomerular filtration rate (eGFR) < 90 ml/min/1.73 m^2^ or markers of kidney damage, and selected those who received a prescription for an RAASi [defined as angiotensin-converting enzyme inhibitors (ACEis) or angiotensin II receptor blockers (ARBs)] during the study period, for whom at least 1 year of data during RAASi treatment was available.

We excluded patients with (1) end-stage renal disease (ESRD) needing dialysis or kidney transplant at baseline (2) no qualifying eGFR measurement, (3) <6 follow-up visits in the study period, and (4) <1 year of observable time during RAASi therapy for renal replacement therapy initiation, loss to follow-up, or death.

For each patient, the follow-up started at baseline (time of enrolment) and continued until the earliest transfer out of the practice, loss to follow-up, dialysis initiation, death, or end of the study period (December 2019).

All the patients were evaluated according to the practice of our center, at baseline, and in follow-up visits at different times according to the degree of reduction in renal function. Age, sex, underlying cause of CKD, and comorbid conditions for all the patients were recorded during baseline visit. Baseline and follow-up visits included blood and urine sample tests (including serum creatinine, serum potassium levels, and 24-h urinary protein excretion amount), eGFR evaluation, clinical examination, vital sign measurements, and interviews for standardized questionnaires.

All laboratory values were measured by the automated, standardized methods used in our hospital. eGFR was estimated using the Chronic Kidney Disease Epidemiology Collaboration (CKD-EPI) equation (19). The MDRD (Modification of Diet in Renal Disease) Study and CKD-EPI equations are the most widely used and thoroughly validated equations for estimation of eGFR: both equations have been validated extensively in subjects with impaired kidney function (eGFR < 60 ml/min/1.73 m^2^), while the CKD-EPI equation has demonstrated improved accuracy for eGFR levels > 60 ml/min/1.73 m^2^ (19). Therefore, since our population study included patients with CKD stages 1–5, we used the CKD-EPI equation. The patients were categorized into the Kidney Disease Improving Global Outcomes (KDIGO) CKD eGFR categories (20): > 90 (stage 1), 89–60 (stage 2), 60–45 (stage 3a), 45–30 (stage 3b), 29–15 (stage 4), and <15 (stage 5) ml/min/1.73 m^2^. Medication and prescription details of the patients were recorded at each visit, such as date of prescription, medication type, strength, dose, and quantity. Moreover, we also evaluated the use of concomitant medications known to affect potassium levels (i.e., diuretics, calcineurine inhibitors, antibiotics such as trimetoprim, NSAIDs [nonsteroidal anti-inflammatory drugs], and SGLT-2 [sodium-glucose transport protein 2] inhibitors).

The study outcomes of interest include: (1) prevalence of patients with CKD treated with RAASis, and distribution of the use of RAASis in different CKD stages; (2) evaluation, in patients with CKD patients with RAASis (overall and by CKD stage), of the incidence of hyperkalemia; (3) proportion of patients with RAASi medication change (permanent cessation, reduction in number of RAASis, or dose reduction) after experiencing incident hyperkalemia.

The study was conducted in accordance with the principles outlined in the Declaration of Helsinki and was approved by a local ethics committee.

### Statistical Analysis

An initial descriptive analysis was performed to investigate baseline characteristics of the study population, incidence of hyperkalemia, and changes in RAASi therapy. Categorical variables were presented as frequency distribution. Normally distributed data were presented as means and standard deviations, whereas non-normally distributed data were reported as median and interquartile range (IQR). Chi squared test was performed to detect differences among proportions. Analysis of variance (ANOVA) and Kruskal-Wallis test were performed to investigate differences in baseline characteristics among CKD stages for normally and non-normally distributed data, respectively.

We analyzed the variables at the time of discontinuation of RAASi and after 8, 16, 24, and 52 weeks (data not shown, available from the corresponding author upon request). Statistical analyses were performed by ANOVA for normally distributed data and Friedman test for nonparametric data. *Post hoc* analyses were performed by Tukey test and Dunn test for parametric and nonparametric data, respectively.

Binary logistic regression was used to estimate the influence of type of RAASi, smoking status, diabetes, heart failure, CKD stage, and sex in modulating the risk of hyperkalemia.

Differences were considered statistically significant when *p*-value was lower than 0.05. Statistical analysis was performed using R version 3.6.1 with R Studio version 1.2.5033.

## Results

**Figure 1:**
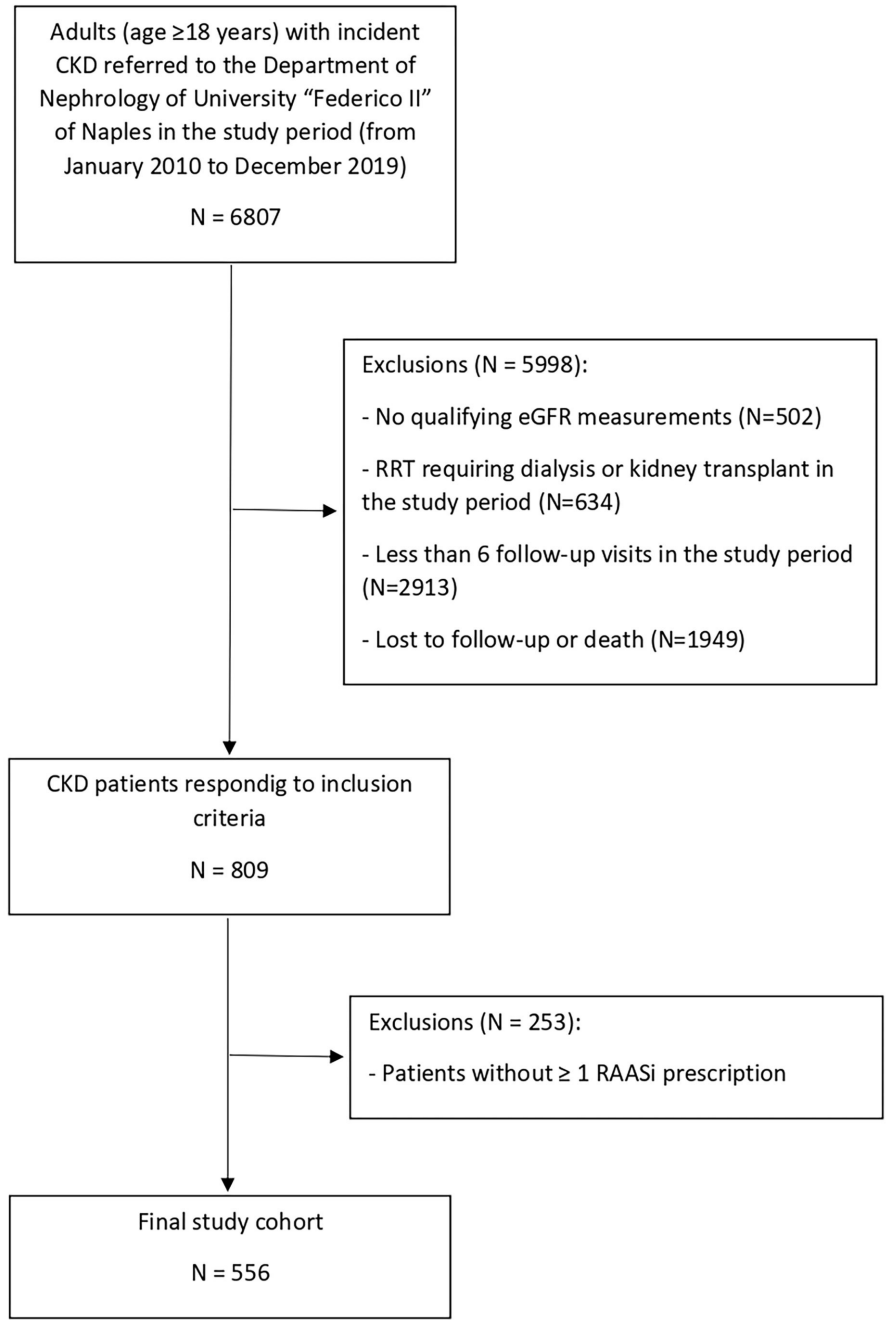
Identification of study cohort. CKD, chronic kidney disease; eGFR, estimated glomerular filtration rate; RAASi, renin-angiotensin-aldosteron system inhibitors; RRT, renal replacement therapy.

We identified 809 adult CKD responding to all inclusion criteria. Of these 809 patients, 556 (68.7%) had ≥ 1 RAASi prescription; thus they were eligible for the study (Figure 1). The main baseline characteristics of the study cohort (overall and by CKD stage) are reported in Table 1. The median age of the cohort was 59.5 years (IQR 26), 46.2% were females, median eGFR was 40.3 ml/min/1.73 m^2^ (IQR 45.5), and median potassium level at baseline was 4.7 mmol/L (IQR.8).

**Table 1:** Baseline characteristics of the study cohort, overall and by CKD stage.

	**Overall**	**CKD stage**					**Test statistic χ^2^**	***p*-value**
		**Stage 1**	**Stage 2**	**Stage 3**	**Stage 4**	**Stage 5**		
Patients	556	57 (10.2)	106 (19.1)	194 (34.9)	114 (20.5)	85 (15.3)	94.558	< 0.0001
**Sociodemographic information**								
*Sex*								
Female	258	30 (52.6)	56 (52.8)	86 (44.3)	43 (37.7)	43 (50.6)	7.0401	0.1338
Male	298	27(47.4)	50 (47.2)	108 (55.7)	71 (62.3)	42 (49.4)		
Age, years (median and IQR)	59.5 (26)	35 (19)	51^*^ (22.5)	65^*^^§^ (25)	67^*^^§^ (24)	60^*^^§^ (18)	123.94	< 0.0001
*Smoking status*								
Smoker	122 (22)	12 (9.8)	7 (5.8)	55 (45)	24 (19.7)	24 (19.7)	29.35	<0.001
Previous smoker	72 (13)	8 (11.1)	24 (33.3)	23 (31.9)	10 (13.9)	7 (9.8)		
Non-smoker	362 (65)	37 (10.2)	75 (20.7)	116 (32.0)	80 (22.1)	54 (15)		
**Laboratory measurements**								
eGFR, mL/min/1.73 m^2^ (median and IQR)	40.3 (45.5)	103.8 (16.1)	74 (13.35)^*^	43.7 (17.8)^*^^§^	22.1 (7.95)^*^^‡^	9 (5.7)^*^^†^	474.41	< 0.0001
Potassium, mmol/L (median and IQR)	4.7 (0.8)	4.4 (0.5)	4.4 (0.5)	4.7 (0.4)^*^^§^	5.1 (0.6)^*^^‡^	4.9 (0.9)^*^^‡^	100.33	< 0.0001
24-h urinary protein excretion (mg/day) (median and IQR)	1,012 (930)	452 (405)	721 (509)^*^	915 (250)^*^	1,712 (890)^*^^‡^	1,516 (560)^*^^†^	34.734	< 0.001
**Comorbid conditions**								
Diabetes mellitus	82	3 (3.7)	4 (4.9)	26 (31.7)^*^^§^	32(39)^*^^§^	17 (20.7)^*^^‡^	32.469	< 0.0001
Heart failure	49	0	8 (7.5)	27 (13.9)^*^	11 (9.6)^*^^§^	3 (3.5)^*^^†^	15.062	<0.01
**RAASi type**								
ACEi	250 (45)	36 (63.1)	53 (50)	84 (43.3)	50 (43.9)	27 (31.8)	29.486	<0.001
ARB	206 (37)	7 (12.3)	30 (28.3)	80 (41.2)	46 (40.3)	26 (31.7)		
ACEi + ARB combination	100 (18)	14 (24.6)	23 (21.7)	34 (17.5)	18 (15.8)	27 (13.9)		

*Values are numbers (percentages) unless stated otherwise*.

*ACEi, angiotensin converting enzyme inhibitors; ARB, angiotensin II receptor blockers; CKD, chronic kidney disease; eGFR, estimated glomerular filtration rate; RAASi, renin-angiotensin-aldosteron system inhibitors; SD, standard deviation*.

* *p < 0.05 compared to stage 1*.

§ *p < 0.05 compared to stage 2*.

† *p < 0.05 compared to stage 2,3, and 4*.

‡ *p < 0.05 compared to stage 2 and 3*.

The prevalence of patients with CKD patients with RAASi was 68.7%; RAASis were equally distributed among stages 2, 3, and 4 (Table 1). In particular, ACEi prescription was less frequent in stages 3, 4, and 5 than in stage 1, whereas ARBs were more frequent in stages 3, 4, and 5 than in stage 1; combination therapy was equally distributed among all the CKD stages (Table 1).

A total of 51 patients with CKD treated with RAASis experienced an episode of hyperkalemia during the follow-up period (cumulative incidence 9.2%; Table 2). Overall, the incidence of hyperkalemia did not differ from stages 1 to stage 3, while it significantly increased in stages 4–5 (Table 2). The rate for hyperkalemia among patients with stage 4–5 CKD was 3-fold higher than in patients with stage 1-2-3 CKD (*p* < 0.0001). Of these 51 patients with hyperkalemia, 40 developed potassium serum levels between 5.1 and 5.5 mmol/L, eight patients between 5.6 and 6 mmol/L, two subjects between 6.6 and 7 mmol/L, and one patient > 7.1 mmol/L.

**Table 2:** Incidence of hyperkalemia and changes in RAASi therapy in the study cohort, overall and by CKD stage.

	**Overall**	**CKD stage**					**Test statistic χ^2^**	***p*-value**
		**Stage 1**	**Stage 2**	**Stage 3**	**Stage 4**	**Stage 5**		
Patients	556	57 (10.2)	106 (19.1)	194 (34.9)	114 (20.5)	85 (15.3)	94.558	< 0.0001
Hyperkalemia	51 (9.2)	0 (0)	3 (2.8)	10 (5.1)	21 (18.4)^*^^§^^‖^	17 (20)^*^^§^^‖^	38.298	< 0.0001
RAASi change	31 (5.6)	0 (0)	2 (1.9)	3 (1.5)	13^*^^§^^‖^ (11.4)	13^*^^§^^‖^ (15.3)	34.692	< 0.0001
Cessation	23 (4.1)	0 (0)	2 (3.5)	3 (1.5)	10 (8.8) ^‖^	8 (9.4) ^*^^§^^‖^	19.236	<0.001
Dose reduction	1 (0.2)	0 (0)	0 (0)	0 (0)	1 (0.9)	0 (0)	3.8842	0.4219
Shift from ACEi + ARB combination to monotherapy	7 (1.3)	0 (0)	0 (0)	0 (0)	2 (1.7)	5 (5.9) ^§^^‖^	19.392	<0.001

*Values are numbers (percentages)*.

*ACEi, angiotensin converting enzyme inhibitors; ARB, angiotensin II receptor blockers; CKD, chronic kidney disease; RAASi, renin-angiotensin-aldosterone system inhibitors; SD, standard deviation*.

* *p < 0.05 compared to stage 1*.

§ *p < 0.05 compared to stage 2*.

‖ *p < 0.05 compared to stage 3*.

^†^*p < 0.05 compared to stage 2,3, and 4*.

^‡^*p < 0.05 compared to stage 2 and 3*.

Moreover, of the 51 patients who experienced incident hyperkalemia, 31 (55.3%) changed RAASi treatment prescription. Among these patients, 23 (74.2%) discontinued RAASi treatment, for one patient (3.2%) was prescribed a reduced dose of these agents, and seven (22.6%) reduced number of RAASi drugs, changing from combination of ACEi + ARB to monotherapy (Table 2). No significant difference in mean serum potassium levels at the time of incident hyperkalemia according to the type of RAASi medication change was observed (data not shown). When assessed according to eGFR categories, RAASi medication changes were more prevalent among those with lower levels of eGFR (Table 2). No patient started hyperkalemia reducing-treatment during the study period. Moreover, patients experiencing hyperkalemia reported a significant reduction of potassium serum levels after RAASi dose reduction or discontinuation (data not shown).

In binary logistic regression analysis, female sex vs. male and status of previous smoker or non-smoker vs. smoker represented protective factors for hyperkalemia. On the contrary, diabetes and CKD stages 3, 4, and 5 vs. stage 1 were found to be risk factors (Table 3). The contribution of proteinuria in the development of hyperkalemia was found to be very small (data not shown).

**Table 3:** Results from binary logistic regression.

**Factor**	**Beta coefficient**	**OR (95% CI)**	**z value**	***p*-value**
Male	ref			
Female	−0.741	0.477 (0.244–0.930)	−2.171	0.0299
Diabetes	0.786	2.195 (1.327–3.632)	3.063	*p* < 0.01
Hearth failure	0.757	2.132 (0.969–4.69)	1.883	0.0597
Age	−0.00333	0.997 (0.983–1.011)	−0.467	0.641
Smoker	ref			
Previous smoker	−1.463	0.231 (0.110–0.489)	−3.839	*p* < 0.001
Non-smoker	−1.193	0.303 (0.186–0.495)	−4.778	p <0.001
CKD stage 1	ref			
CKD stage 2	0.4178	1.519 (0.549–4.197)	0.805	0.4205
CKD stage 3	1.290	3.633 (1.426–9.250)	2.705	*p* < 0.01
CKD stage 4	2.367	10.665 (3.975–28.613)	4.701	*p* < 0.01
CKD stage 5	1.905	6.719 (2.470–18.276)	3.732	*p* < 0.01

*CI, confidence interval; CKD, chronic kidney disease; OR, odds ratio*.

Finally, no correlation was found between use of concomitant medications affecting potassium levels and development of hyperkalemia (data not shown).

## Discussion

In our CKD-population-based cohort study, we observed an overall hyperkalemia incidence of 9.2% in over 556 patients with CKD s treated with RAASis, which did not significantly increase from stage 1 to 3, while it significantly augmented from stages 3 to 5.

Among those who experienced incident hyperkalemia, most patients changed RAASi medication therapy (permanent cessation, reduction in number of RAASis, or dose reduction) (Table 2).

Several articles have reported that RAASis are underutilized in patients with CKD, and that hyperkalemia is the major barrier for their initiation or sustained treatment. In a recent report of Shiriziad et al. that examined variables associated with prescription of renin-angiotensin system blockers in patients with CKD, the authors reported that 36% of patients with moderate to severe CKD and indication for RAASi therapy were not prescribed renin-angiotensin system blockade, and that the most common documented reasons were hyperkalemia and history of acute kidney injury (21). RAASi discontinuation in the presence of a clear RAASi indication has been recently associated with adverse outcomes (13, 22, 23). Epstein et al., in a comprehensive analysis of a large database of electronic medical records (> 7 million patients), showed that maximum RAASi doses were prescribed in 19 to 26% of patients, that a substantial proportion of patients had changes in their dose following a hyperkalemia event, and that patients who were on submaximum or discontinued RAASis had worse cardiorenal outcomes and higher mortality than patients on maximum dose, irrespective of comorbidity status or patient age (13).

It is, therefore, important to determine the prevalence and risk factors of hyperkalemia secondary to RAASi therapy, and the relationship between hyperkalemia and treatment interruptions/cessations.

Consistently with the results of the current literature, our study confirms that history of CKD (stages 3–5) is associated with elevated risk of hyperkalemia as well as with RAASi treatment modifications (14, 24). Overall, the adjusted rate for hyperkalemia among patients with stage 4–5 CKD was 3-fold higher compared with patients with eGFR > 60 ml/min/1.73 m^2^.

Reported percentages of RAASi therapy discontinuation due to hyperkalemia from randomized trials in patients with CKD are limited. One study reported a dose reduction or therapy discontinuation rate of 47% (13); another population-based study reported RAASi dose reduction and discontinuation prevalence of 1.9 and 35.2% (18), respectively, among patients with CKD and eGFR <30 ml/min/1.73 m^2^ (*n* = 346). In another article, a more recent study on 20,184 patients with CKD, 46.6% of them showed changes in their RAASi therapy (discontinuation: 36.6% and dose reduction: 10%) (25). Our results are consistent with these data and suggest that RAASi medication change is relatively common in routine clinical settings among patients with CKD who experience hyperkalemia. Taken together, these findings provide strong evidence that hyperkalemia is a critical factor limiting RAASi therapy in patients with CKD, and suggest that these patients might be suitable potential targets for long-term potassium-lowering treatments. In particular, available randomized controlled trials have shown that newer potassium-reducing therapies, such as sodium zirconium cyclosilate (SZC) and patiromer, have effectively and safely reduced serum potassium levels and maintained long-term normokalemia in patients with CKD treated with RAASis (26–31). Recent clinical studies suggest that these newer potassium binders may facilitate optimization of RAASi therapy (32). Finally, a virtual panel recently met with the purpose of developing practical solutions in the identification and management of hyperkalemia in patients with CKD and/or heart failure. The panel developed an algorithm, the Proposed Diagnostic Algorithm for Hyperkalemia Treatment in the Acute Care Setting/Chronic Care, and agreed that patiromer appears to be a viable option for the management of hyperkalemia in patients with CKD experiencing hyperkalemia (33). However, its safety and efficacy over a longer period remain to be ascertained with real-life experience.

As with any retrospective observational study, our study has some limitations that should be considered: at first, we could not select only new-user patients due to the impossibility to exactly define the start date of RAASi therapy already present at first visit. Moreover, we were limited in our ability to assess for possible temporary periods without being under RAASi therapy, for the analysis of sodium and potassium dietary intake, and for the use of concomitant medications including those known to affect potassium serum levels. Moreover, we do not have bicarbonate values for our patients, so we could not investigate for a possible correlation between hyperkalemia and metabolic acidosis, in particular for patients with eGFR < 30 ml/min. Finally, we did not include a “control” group of patients with CKD receiving other antihypertensive medications to compare outcomes or incidence of hyperkalemia.

In conclusion, in this study on a large cohort of adults RAASi users with CKD, the incidence of hyperkalemia was frequently observed, and rates increased with worsening levels of renal function from stages 1 to 3. RAASi medication change following an episode of hyperkalemia occurred in almost half of the patients after experiencing hyperkalemia.

## Data Availability Statement

The raw data supporting the conclusions of this article will be made available by the authors, without undue reservation.

## Ethics Statement

The studies involving human participants were reviewed and approved by Carlo Romano. Written informed consent to participate in this study was provided by the participants' legal guardian/next of kin.

## Author Contributions

AP had the original idea. ER, IC, MAn, MP, MR, and MAm took in care the patient. PB performed the statistic evaluation. AP, ER, and IC wrote the paper. All authors have read and approved the final version of the manuscript.

## Conflict of Interest

The authors declare that the research was conducted in the absence of any commercial or financial relationships that could be construed as a potential conflict of interest.

## Publisher's Note

All claims expressed in this article are solely those of the authors and do not necessarily represent those of their affiliated organizations, or those of the publisher, the editors and the reviewers. Any product that may be evaluated in this article, or claim that may be made by its manufacturer, is not guaranteed or endorsed by the publisher.
